# Hawaiʻi Suicide Rates by Occupation 2013–2023

**DOI:** 10.3390/ijerph23040422

**Published:** 2026-03-27

**Authors:** Thao N. Le, Daniel J. Galanis

**Affiliations:** 1 Family Consumer Sciences, College of Tropical Agriculture & Human Resilience, University of Hawaiʻi Mānoa, 110 Miller Hall, 2515 Campus Road, Honolulu, HI 96822, USA; 2 Emergency Medical Services & Injury Prevention System Branch, Hawaiʻi State Department of Health, 3675 Kilauea Ave, Honolulu, HI 96816, USA

**Keywords:** suicide, mortality, occupation, industry, mental health, Hawaiʻi

## Abstract

**Highlights:**

**Public health relevance—How does this work relate to a public health issue?**

- Identifies high-risk vulnerable groups by revealing occupational disparities/differences in suicide rates like construction, farming, and the arts/entertainment.
- Allows public health officials to develop targeted mental health prevention/intervention strategies to address the unique stressors of different high-risk occupations.

**Public health significance—Why is this work of significance to public health?**

- Provides data and empirical evidence to help public health agencies allocate resources more effectively to address multi-factorial risks.
- Highlights the continued need for states to capture epidemiological and surveillance data/statistics on injuries, including suicide.

**Public health implications—What are the key implications or messages for practitioners, policy makers and/or researchers in public health?**

- Findings underscore the critical importance for states to maintain robust surveillance data and statistics on injuries and suicide to inform ongoing prevention and intervention efforts.
- The study provides empirical data that allows public health agencies to allocate suicide prevention resources more effectively by targeting certain populations of high-risk occupations.

**Abstract:**

The suicide rate in the U.S. has increased in the last two decades despite continued efforts to mitigate risks. This study explored potential variability in suicide rates by occupation in Hawaiʻi by analyzing 2430 death certificates from 2013 to 2023. Of these, 1988 suicide deaths occurred among individuals aged 20 to 64 years old, who constituted the study sample. Suicide death was identified using ICD-10 underlying cause of death codes X60–X84 and Y87. Occupations were coded using the 1990 Census Bureau classification scheme, and nearly all records included open-text occupation information, supplemented by business/industry descriptions. The numerator was calculated as the 11-year suicide total by occupation; the denominator was based on the annual PERWT population estimate (U.S. Census/IPUMS data) over the same 11-year period. The mean victim age was 41, and 78% were males, with notable variation by occupation. Occupations with the highest suicide rates included carpenters, construction, farmers and fishers, musicians and artists, as well as landscapers and groundskeepers, comparable to national and a few other state-specific reports. The findings are limited, constrained by potential confounding factors and the multi-factorial nature of risk of suicide, imprecise numerator and denominator match, as well as the influence of retirees in the computation of rates.

## 1. Introduction

Suicide remains a major public health concern in the United States, with national rates increasing substantially over the past two decades [1,2]. Suicide currently surpasses motor vehicle crashes and drownings and is the second-leading cause of fatal injuries nationally [3], and in Hawaiʻi, it is the leading cause for 15- to 29-year-olds and the second-leading cause of injury mortality for 30- to 74-year-olds [4]. Research consistently shows that risk is not uniform across the population, and occupational context has emerged as a meaningful correlate of suicide mortality. National and state-level studies have identified elevated suicide rates in sectors such as construction, agriculture and extraction, military service, and the arts, while other fields are associated with lower risk [5,6]. Farmers, for instance, often rank among the highest due to experiencing chronically high levels of uncertainty (e.g., pests, weather, and market prices), as well as isolation and access to lethal means (e.g., firearms and pesticides) [7,8]. Studies have also identified suicidal ideation as being significantly influenced by occupational factors [9], including low job control, lack of supervisory support, or job insecurity; job strain and long working hours; manually demanding labor affecting physical health; high-stress environments such as repeated exposure to death or injury; and access to lethal means [10].

Understanding variation in suicide rates by occupation is particularly important for states such as Hawaiʻi, where unique labor market structures, demographic profiles, rich cultural diversity, and rural–urban differences may shape both risk and prevention opportunities. Examining occupation-specific patterns can inform tailored workplace interventions, resource allocation, and policy responses. Thus, a periodic review of suicide rates across occupations is warranted. The primary aim of this study is to quantify suicide fatality rates across detailed occupational groupings in Hawaiʻi and to examine age- and sex-specific patterns that may guide targeted prevention efforts.

## 2. Materials and Methods

The study sample comprised selected elements from 1988 Hawaiʻi electronic death certificate records for suicide victims, 20 through 64 years of age, from 2013 to 2023. Decedents who were 65 years or older (*n* = 433) were excluded to minimize skewing the suicide rate (e.g., retirees) due to data unreliability regarding usual occupation and suicide death. Retirement status is not routinely recorded on death certificates in Hawaiʻi, or if indicated, may not specify their former or usual occupation. In addition, nine other records of 20- to 64-year-old decedents were also excluded based on open text describing their usual occupation as being retired (e.g., “teacher retired”). Suicides were identified using ICD-10 underlying cause-of-death codes X60–X84 and Y87.0. Nearly all records (98%) included open-text information describing the decedent’s usual occupation, supplemented by a separate open-text field for the kind of business or industry to aid clarification.

Occupation coding relied on the 1990 U.S. Census Bureau occupational classification scheme. This scheme was based on a reference file that was constructed from 95,889 Hawaiʻi death certificates issued between 1999 and 2012, which yielded 19,177 unique combinations of occupation text and corresponding occupation codes assigned by trained nosologists. Business and industry text was used to resolve cases where a single occupation description mapped to multiple codes; remaining ambiguities were resolved using the most frequent code assignments. We were able to match verbatim approximately 80% of suicide records to the reference file and, therefore, to the nosologist-assigned occupation codes. The remaining records were coded following manual review of occupation and business or industry text. Occupation codes could not be assigned to 103 records (5.2%) due to missing or nonspecific information.

Occupation codes were analyzed individually (e.g., carpenters) or aggregated into broader groupings to achieve minimum cell sizes (*n* ≥ 30) for mortality rate estimation, following the IPUMS 1990 occupation classification scheme [11]. Twenty-five categories were ultimately constructed, based on single or grouped occupations, including “other specified occupations.” Rates were not calculated for the 38 decedents in this latter group, as it included disparate occupations (e.g., animal care, seamstress, etc.) that did not clearly fit in the other categories and numbered too few for independent analyses.

The denominators for rate calculations were taken from IPUMS, which provided annual age- and sex-specific estimates of the number of people employed in the coded occupations over the study period. IPUMS data were first limited to the 20- to 64-year age range of the study, and then by the 166 occupation codes describing the twenty-three occupation groupings (after exclusion of the “other occupational groupings” and unemployed/never worked). For crude rate calculation, the numerator was the 11-year sum of the estimated number of people employed in a given occupation code(s), using the PERWT variable (U.S. Census/IPUMS). Age-adjusted rates were also calculated across four age groups: 20–29 years, 30–39, 40–49, and 50–64. The U.S. 2000 age distribution for that age range was used as the standard [12]. Age- and sex-specific rates were also calculated, using corresponding IPUMS data groupings. Differences in the mean ages among occupation groups were assessed with the Tukey HSD test for multiple comparisons. Crude mortality rates were compared by Fisher’s exact test, and age-adjusted rates by Z-tests. Statistical significance was determined at the 0.05 level. All data analyses were conducted using JMP statistical software (JMP, V.14.2.0, SAS Institute, Cary, NC, USA, 1989–2019).

The University of Hawaiʻi Office of Research Compliance, Human Studies Program provided approval for human research, protocol number 2024-00841 on 7 February 2025. The first author also submitted a data request approval to the State of Hawaiʻi Department of Health’s Institutional Review Committee for access to vital records (i.e., death certificates) for research purposes. Approval was granted on 14 April 2025 for the release of information from vital records without informed consent.

## 3. Results

### 3.1. Suicide Rates by Major Occupational Category

The average age of decedents was 41 years old (*SD* = 13), with substantial variation across occupational groups (Table 1). Individuals employed by the military were the youngest on average (31 years), as were cooks and other restaurant workers, construction laborers, and farmers and fishers. Mean ages were lower for decedents in these groups than for those in the management and healthcare occupations.

Males accounted for more than three-quarters of all deaths (*n* = 1554; 78%) and 93% or more of deaths in 11 occupational groups, including the military, mechanics, drivers, public safety and first responder workers, construction workers, and farmers and fishers. Females constituted a slight majority among decedents employed in healthcare (54%) and nearly half (48%) of the victims in administrative or clerical support.

**Figure 1:**
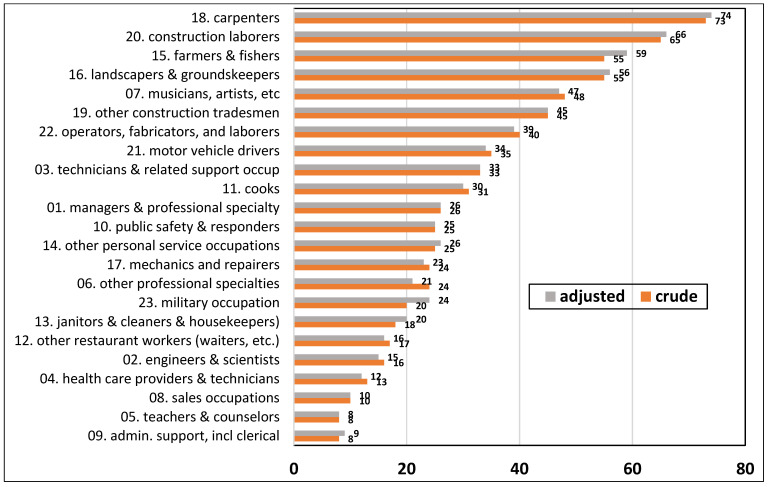
Crude and age-adjusted suicide fatality rates (/100,000 workers) by decedent’s usual occupational category, Hawaiʻi 2013–2023.

Estimated crude and age-adjusted suicide fatality rates varied widely across occupational groups (Table 2; Figure 1). Within most groups, crude and adjusted rates were similar, with the notable exception of military personnel, which possibly reflects denominator effects from retirees. For the ‘all other occupations’ and ‘unemployed/never’ worked categories, there is no accurate denominator data (i.e., the number of workers); thus, crude rate and age-adjusted rates could not be calculated. Note also that the standard U.S. death certificates do not routinely collect information on decedent retirement status; in fact, there are specific instructions not to describe the decedent’s occupation as ‘retired.’

Carpenters had the highest crude and age-adjusted fatality rates, followed by construction laborers, farmers and fishers, landscapers and groundskeepers, and musicians and artists. These five occupational groups had age-adjusted rates of 46 per 100,000 or higher, which are higher than those of the 14 groups with adjusted rates under 30 deaths per 100,000. The lowest rates were observed in administrative support and clerical occupations. This category primarily included general office clerks (17% of 69 victims), stock and inventory clerks, and customer service representatives. Low rates were also observed among education services (e.g., teachers), sales workers, healthcare providers, and engineers and scientists.

### 3.2. Occupational Groups with Highest Suicide Rates

Fatality rates were further examined by age group for the five occupational groupings with the highest mortality rates (Table 3). Analyses were limited to males, who accounted for nearly all deaths in these occupational groups (94%, or 255 of 272 decedents). Because certain age groups had cell sizes less than 10, the respective table and figure only display aggregate information.

**Figure 2:**
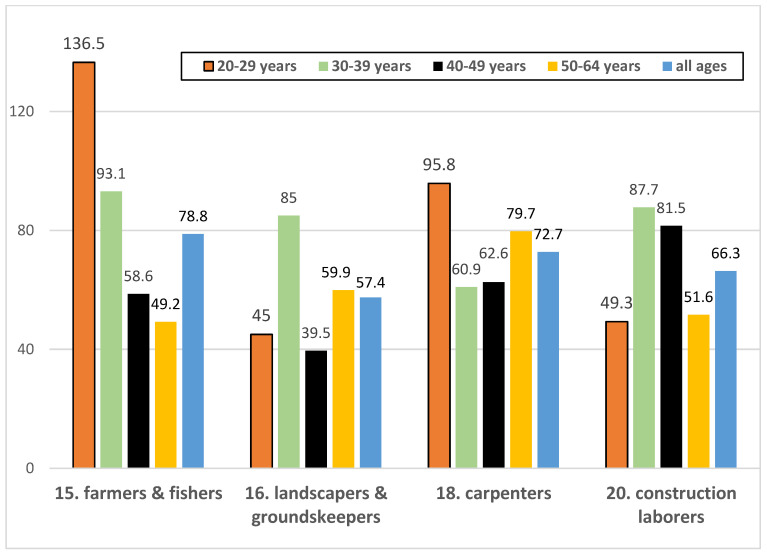
Crude suicide fatality rates among males by select occupational groups and age groups, Hawaiʻi 2013–2023.

Mortality rates were highest for farmers and fishers, although there were no statistically significant differences between any of the five groups (*z*-value of 1.70 or less for all age-adjusted rate comparisons). Crude mortality rates progressively decreased across the age range among farmers and fishers, with a significantly higher rate among 20- to 39-year-olds compared to 50- to 64-year-olds (*p* = 0.0129 for Fisher’s exact test). There were no clear associations between age and crude mortality rates for the other three occupational groups, and there were too few deaths (*n* = 21) among musicians and artists for age-stratified comparisons (hence, Figure 2 only displays four groups).

## 4. Discussion

The findings of this study are largely consistent with prior U.S. research demonstrating substantial variation in suicide risk by occupation, age, and sex, and that these patterns are persistent across time and geography. The predominance of males and the elevated rates observed in construction, agriculture, fishing, and arts-related occupations closely parallel national patterns reported by the CDC [5], as well as earlier state-based studies [6], which consistently identify construction and extraction, farming and fishing, installation and repair, and arts and entertainment as high-risk occupational groups. The one notable exception is mining and extraction, which is absent in Hawaiʻi. Nevertheless, the younger average age of decedents in military, construction, and agriculture/food service occupations aligns with studies highlighting elevated suicide risk among younger and working-age adults in physically demanding or lower-skilled occupations, seasonal labor, and lower socioeconomic status [13,14,15]. Occupation reflects ‘usual occupation,’ and can reflect past employment.

The exceptionally high age-adjusted rates observed for military-related occupations in this study underscore methodological challenges also noted in the previous literature [16], particularly denominator effects arising from potentially retired individuals being included in the numerator but comprising a relatively small proportion of the workforce estimates. The denominator often only counts active-duty personnel or those currently employed in a specific sector, resulting in potentially biased estimations. Reserve and guard members could bias this too due to potential nuances of healthcare access due to their reserve and guard status.

The elevated risks among carpenters, construction laborers, and farmers and fishers support evidence from national surveillance indicating heightened suicide risk in manual labor and resource-based occupations [5]. In contrast, lower rates among administrative, education, healthcare, and sales occupations are consistent with prior findings suggesting protective effects associated with higher job stability, access to benefits, or educational attainment [15]. Specifically, recent work by Messias et al. reinforces this interpretation, demonstrating that educational attainment may confound observed occupational gradients in suicide risk [15]. Collectively, these results support existing evidence while emphasizing the importance of contextual interpretation, reflecting a complex interaction of work conditions, demographic composition, access to lethal means, access to healthcare and mental health services, and broader socioeconomic factors rather than occupation alone.

Despite the noteworthy findings, there are several study limitations or considerations that need to be acknowledged. First, the calculation of suicide rates by occupation is sensitive to the choice of the data source used for the workforce denominator. Estimates derived from the U.S. Census Bureau, the Bureau of Labor Statistics (BLS), and the American Community Survey (ACS) may differ because of methodological differences in how workers are defined and counted. For example, BLS employment figures for agricultural producers typically include only formally employed workers (i.e., excluding self-employment), whereas the U.S. Department of Agriculture estimates may also capture part-time or self-identified farmers, yielding markedly different population counts. Such discrepancies can lead to over- or underestimation of occupation-specific suicide rates.

Use of the U.S. Census/IPUMS [17] [https://usa.ipums.org/usa/volii/occ1990.shtml, accessed on 5 December 2025] mitigates these limitations by providing updated, age- and sex-specific workforce estimates derived using a consistent methodology across a comprehensive range of occupations. As mentioned earlier, trained nosologists reviewed death certificates during 1999–2012, which provided a reference for mapping free-text occupations to standardized codes. The IPUMS OCC1990 field, developed using Census Bureau double-coded samples and refined by the Bureau of Labor Statistics, supports consistent alignment of occupations across census years. Yet, although based on historical codes by nosologists, the reliability of the assigned occupational codes remains unknown. Further, the IPUMS data are also estimates, nonetheless, and were observed to vary widely across the 11-year study period. Use of the IPUMS data did allow for age and sex stratifications of interest across occupation-specific suicide rates better than other potential data sources.

A second consideration concerns the influence of retirees in the suicide fatality rate computations. Occupational suicide rates can be distorted when a potential non-negligible share of decedents is retired, while the corresponding workforce denominator includes relatively few older active workers. Because we do not have information on the retirement status of decedents (i.e., retirement information is not captured in the death certificates), including those employed in the military, the age distribution of military victims, for instance, can be potentially skewed toward younger ages, lending plausibility to the possibility that “military” is not the ultimate usual occupation for many suicide decedents who were in the military earlier in their lives. Although the military group was not included in the analyses summarized in Figure 2, we conducted an exploratory analysis to assess possibly high rates among “military” decedents in younger ages (i.e., the not retired age range). The results did not suggest notably high rates; however, as the “military” group had the 16th highest rate of 24 occupation groupings for 20–39 year-olds, and the 14th highest for 40–64 year-olds. Nevertheless, there are conceptual questions about whether occupational suicide risk persists after retirement or diminishes once the occupational exposure ends. If risk is primarily associated with active employment, attributing suicides among retirees to prior occupations may overstate associations. Given these uncertainties, restricting analyses to decedents younger than 65 years may provide more valid and interpretable estimates of occupation-related suicide risk, specifically for these occupations.

Finally, the multi-factorial nature of suicide risks cannot be discounted. Observed associations were adjusted only for age and sex, leaving many potential unaccounted confounders. Factors such as access to healthcare and mental health services, social isolation, underlying mental health conditions, financial stress, and access to lethal means may substantially influence suicide risk but cannot be evaluated using death certificate data, specifically a summary of usual occupation alone. The dataset includes information only on suicide decedents, limiting the ability to assess how these characteristics compare with the general population. Future studies could improve interpretation by incorporating population-level comparisons on factors such as rurality, educational attainment, and socioeconomic status to better contextualize occupation-specific suicide fatality rates.

Despite these limitations, the findings of this study have important implications for suicide prevention efforts, highlighting the need for targeted, occupation-specific interventions, and even sex/gender and age-specific interventions, such as among farmers. Elevated suicide rates in construction, agriculture, fishing, and the arts, as well as certain service occupations, suggest that workplaces in these sectors are critical settings for prevention. Interventions should prioritize male-dominated and younger workforces, incorporate routine mental health screening, improve access to care, and address job-related stressors such as physical strain, job insecurity, and isolation. Differences in age patterns and likely method availability underscore the importance of tailoring strategies by occupation, including lethal means safety (access to lethal means), peer support programs, supervisor training, and organizational cultural shifts such as attitudes toward health and mental health, including access to any type of mental and physical healthcare holistically. Both target-specific (e.g., increasing awareness and access to resources) and broad workplace-based prevention strategies (e.g., improving working conditions), complemented by population-level approaches (e.g., reducing stigma), may reduce occupational disparities in suicide risk.

In Hawaiʻi, existing evidence-based strategies such as lethal means safety have been a focus of suicide prevention, particularly for rural residents and professions disproportionately affected by lethal means when it comes to suicide death. Specifically, hanging prevention has been a priority for the Hawaiʻi Governor’s Challenge to Prevent Suicide Among Service Members, Veterans, and their Families [18]. Hawaiʻi does not have an institutionally sustained, funded, and implemented Zero Suicide Initiative (Zero Suicide—Suicide Prevention Resource Center); as such, developing and expanding lethal means safety along with the Zero Suicide framework with a focus on healthcare (e.g., primary and rural health) could especially be beneficial for the overall prevention efforts to address suicide disparities.

Notably, Hawaiʻi is one of the first states to implement statewide suicide prevention trainings (i.e., gatekeeper trainings) and has extensive gatekeeper training offerings, including but not limited to Mental Health First Aid (MHFA), Question, Persuade, and Refer (QPR), Connect, Sources of Strength, Applied Suicide Intervention Skills Training (ASIST), safeTALK, American Foundation for Suicide Prevention (AFSP) “Talk Saves Lives,” Suicide Prevention 1010, and Be Sensitive Be Brave. Hawaiʻiʻs State Department of Health is working on revamping the prevention messaging through the Positive Community Norms model with many cross-agency partners, and considering prevention/interventions that allow for cultural consideration, and are collaborative and upstream in approach.

With respect to high-risk occupations such as farmers, the efforts of the Seeds of Wellbeing—College of Tropical Agriculture & Human Resilience (SOW-CTAHR) [19] are an example of a stress and suicide prevention programmatic effort that specifically targets farmers and other agricultural producers in Hawaiʻi, emphasizing a peer support model and incorporating QPR trainings that are farmer-specific through AgriSafe [20] Another example is the AFSP Hawaiʻi Chapterʻs hosting of their “Hike for Hope” with a focus on the construction industry for a second year, while Hawaiʻi (Big Island) is developing their own community-based suicide prevention training videos with local scripts, examples, interventions, and train-the-trainer curriculums.

## 5. Conclusions

This study demonstrates substantial variation in suicide risk by occupation, age, and sex/gender, reinforcing occupation as an important contextual factor in suicide mortality and a public health suicide prevention strategy. For Hawaiʻi, the elevated rates among construction, arts and entertainment, and agriculture and fishing were consistent with prior research. Lower rates in administrative, education, and healthcare occupations further underscore heterogeneity across occupational categories. Together, these findings support the need for careful interpretation of occupation-specific suicide rates and for targeted, evidence-based prevention strategies tailored to high-risk occupational groups.

## Figures and Tables

**Table 1:** Demographics of suicide decedents by occupational category, Hawaiʻi 2013–2023.

Occupational Grouping	Number (% of Total)	Age (±SD)	Sex (Percent Male)
01. managers and professional specialty	221 (11.1%)	45 (+12)	77%
02. engineers and scientists	35 (1.8%)	45 (+12)	77%
03. technicians and related support	41 (2.1%)	44 (+12)	93%
04. healthcare providers and technicians	72 (3.6%)	48 (+11)	46%
05. teachers and counselors	37 (1.9%)	46 (+14)	62%
06. other professional specialties	46 (2.3%)	50 (+9)	54%
07. musicians and artists, etc.	32 (1.6%)	41 (+12)	66%
08. sales occupations	77 (3.9%)	44 (+14)	62%
09. admin. support, including clerical	69 (3.5%)	38 (+12)	52%
10. public safety and first responders	60 (3.0%)	41 (+12)	98%
11. cooks	70 (3.5%)	37 (+12)	87%
12. other restaurant workers (waiters, etc.)	58 (2.9%)	36 (+13)	71%
13. janitors, cleaners and housekeepers	50 (2.5%)	41 (+13)	80%
14. other personal service occupations	65 (3.3%)	43 (+12)	57%
15. farmers and fishers	45 (2.3%)	37 (+13)	98%
16. landscapers and groundskeepers	58 (2.9%)	42 (+13)	97%
17. mechanics and repairers	48 (2.4%)	44 (+14)	100%
18. carpenters	66 (3.3%)	43 (+13)	97%
19. other construction tradesmen	106 (5.3%)	42 (+13)	99%
20. construction laborers	71 (3.6%)	39 (+11)	99%
21. motor vehicle drivers	69 (3.5%)	44 (+12)	100%
22. operators, fabricators, and laborers	83 (4.2%)	40 (+14)	98%
23. military occupation	96 (4.8%)	31 (+9)	94%
24. all other specified occupations	38 (1.9%)	42 (+12)	71%
25. unemployed and never worked	272 (13.7%)	37 (+13)	59%
26. unknown	103 (5.2%)	48 (+11)	79%
Total	1988	41 (+13)	78%

**Table 2:** Suicide rates by decedent’s usual occupational category, Hawaiʻi 2013–2023.

Occupational Grouping	Number	Estimated Number of Workers	Crude Rate (/100,000 Workers)	Age-Adjusted * Rate (/100,000 Workers)
01. managers and professional specialty	221	841,874	26.3	26.3
02. engineers and scientists	35	224,749	15.6	15.4
03. technicians and related support	41	123,995	33.1	32.7
04. healthcare providers and technicians	72	569,786	12.6	12.3
05. teachers and counselors	37	447,071	8.3	7.9
06. other professional specialties	46	190,890	24.1	20.7
07. musicians and artists, etc.	32	66,761	47.9	46.6
08. sales occupations	77	782,066	9.8	9.9
09. admin. support, including clerical	69	844,911	8.2	8.6
10. public safety and first responders	60	235,741	25.5	25.4
11. cooks	70	229,017	30.6	30.1
12. other restaurant workers (waiters, etc.)	58	348,255	16.7	16.4
13. janitors, cleaners, and housekeepers	50	273,387	18.3	20.3
14. other personal service occupations	65	262,798	24.7	25.8
15. farmers and fishers	45	81,883	55.0	58.8
16. landscapers and groundskeepers	58	105,962	54.7	55.8
17. mechanics and repairers	48	198,944	24.1	23.4
18. carpenters	66	90,437	73.0	73.7
19. other construction tradesmen	106	236,741	44.8	44.8
20. construction laborers	71	109,924	64.6	66.1
21. motor vehicle drivers	69	199,982	34.5	33.8
22. operators, fabricators, and laborers	83	209,951	39.5	38.9
23. military occupation	96	482,238	19.9	23.9
24. all other specified occupations	38			
25. unemployed and never worked	272			
26. unknown	103			
Total (excluding 24, 25, 26)	1575	7,157,363	22.0	21.9

* Adjusted for four age groups (20–29 years, 30–39 years, 40–49 years, and 50–64 years), against the U.S. 2000 standard distribution.

**Table 3:** Crude suicide fatality rates among males in Hawaiʻi, for select occupational groups, 2013–2023.

Occupational Grouping	Number of Deaths	Estimated Number of Workers	Crude Rate (/100,000 Workers)	Age-Adjusted Rate (/100,000 Workers)
07. musicians, artists, etc.	21	38,116	55.1	59.5
15. farmers and fishers	44	55,803	78.8	82.5
16. landscapers and groundskeepers	56	97,545	57.4	57.7
18. carpenters	64	88,034	72.7	74.0
20. construction laborers	70	105,657	66.3	68.3

## Data Availability

The data and datasets generated during this study are not publicly available due to ethical restrictions and the sensitive nature of participant data.
